# Moderate Sedation or Deep Sedation for ERCP: What Are the Preferences in the Literature?

**DOI:** 10.3390/life14101306

**Published:** 2024-10-15

**Authors:** Giuseppinella Melita, Vincenzo Francesco Tripodi, Socrate Pallio, Endrit Shahini, Alessandro Vitello, Emanuele Sinagra, Antonio Facciorusso, Anna Teresa Mazzeo, Arup Choudhury, Jahnvi Dhar, Jayanta Samanta, Marcello Fabio Maida

**Affiliations:** 1 Human Pathology Department, University of Messina, 98124 Messina, Italy; giuseppinella.melita@unime.it; 2 Anesthesia and Intensive Care, Human Pathology Department, University of Messina, 98124 Messina, Italy; annateresamazzeo@unime.it; 3 Clinical and Experimental Medicine Department, University of Messina, 98124 Messina, Italy; socrate.pallio@unime.it; 4 Gastroenterology Unit, National Institute of Gastroenterology, IRCCS “Saverio de Bellis”, Castellana Grotte, 70013 Bari, Italy; endrit.shahini@irccsdebellis.it; 5 Gastroenterology and Endoscopy Unit, S. Elia-Raimondi Hospital, 93100 Caltanissetta, Italy; alessandrovitello86@gmail.com; 6 Gastroenterology and Endoscopy Unit, Fondazione Istituto San Raffaele Giglio, 90015 Cefalù, Italy; emanuelesinagra83@googlemail.com; 7 Gastroenterology Unit, Department of Medical and Surgical Sciences, University of Foggia, 71100 Foggia, Italy; antonio.facciorusso@virgilio.it; 8 Gastroenterology Unit, Post Graduate Institute of Medical Education and Research, Chandigarh 160012, India; drarupc@gmail.com (A.C.); jahnvi3012@gmail.com (J.D.); dj_samanta@yahoo.co.in (J.S.); 9 Department of Medicine and Surgery, University of Enna “Kore”, 94100 Enna, Italy; 10 Gastroenterology Unit, Umberto I Hospital, 94100 Enna, Italy

**Keywords:** endoscopic retrograde cholangiopancreatography, anesthesia, moderate sedation, deep sedation, literature review

## Abstract

One of the most essential procedures for individuals with biliopancreatic disorders is endoscopic retrograde cholangiopancreatography (ERCP). It is based on the combination of endoscopy and radiology to study the biliopancreatic ducts and apply therapeutic solutions. ERCP is currently used to treat choledocholithiasis with or without cholangitis, as well as pancreatic duct stones, benign bile, and pancreatic leaks. On the other hand, ERCP is an unpleasant procedure that must be conducted under anesthetic (moderate sedation, deep sedation, or general anesthesia). With procedures becoming more challenging, the role of anesthesia in ERCP has become even more relevant, and the use of general anesthesia has become better defined. In the last decades, many drugs have been used and some new drugs, like dexmedetomidine, have been recently introduced for sedation or anesthesia during ERCP. Moreover, the scientific community is still divided on the level of sedation to be applied, as well as on appropriate airway management. We therefore performed a narrative review of the literature to assess currently available anesthetic medications for elective ERCP and evidence supporting their effectiveness.

## 1. Introduction

Endoscopic retrograde cholangiopancreatography (ERCP) is a highly effective procedure for patients with biliopancreatic disorders. It is based on the combination of endoscopy and radiology to study the biliopancreatic ducts that carry bile and pancreatic juice to the small bowel. Over time, ERCP has progressively evolved from a diagnostic to a therapeutic procedure in both hepatobiliary and pancreatic diseases [1,2]. The use of ERCP for diagnostic purposes has been supplanted by non-invasive methods such as magnetic resonance imaging (MRI cholangiopancreatography) and endoscopy ultrasound (EUS).

At present, ERCP is predominantly used for treating choledocholithiasis, whether accompanied by cholangitis or not. It is also employed for addressing pancreatic duct stones and benign and malignant strictures, as well as bile and pancreatic leaks.

With procedures becoming more challenging, the role of anesthesia in ERCP has become even more relevant, and the use of general anesthesia has become better defined.

On the other hand, ERCP is an extremely specialized procedure that cannot be performed without adequate anesthesia [3]. The examination lasts approximately 30 to 60 min, depending on the complexity of the procedure and any treatments that the operator may decide to carry out. The procedure is, therefore, performed by moderate sedation, deep sedation, or general anesthesia with hospitalization.

**Figure 1:**
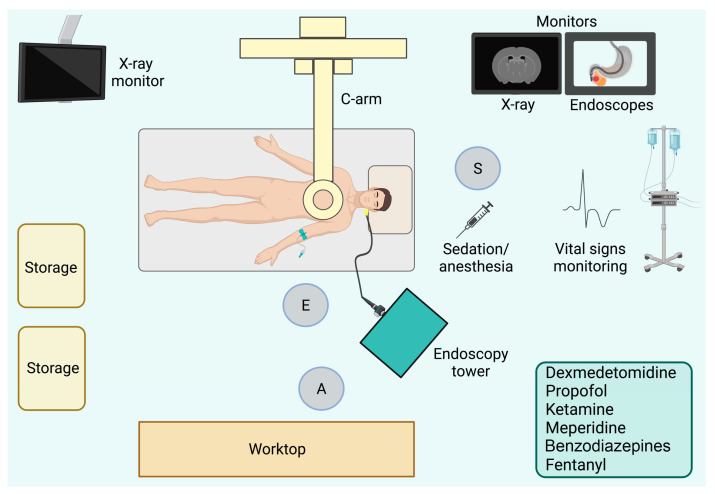
“ERC Floor Plan”. E: Endoscopist. A: Assistant. S: Anesthetist.

In the last couple of decades, many drugs have been used and some new drugs have been recently introduced for sedation or anesthesia during ERCP [3,4]. Moreover, the scientific community is still divided on the level of sedation to be applied during an ERCP procedure. The collaboration between members of the operating team, and in particular between the endoscopist and anesthetist, is essential to reduce perioperative risks and for a successful procedure [Figure 1].

We therefore performed a narrative review to assess the efficacy and safety of currently available anesthetic medications for elective ERCP procedures and evidence supporting moderate sedation and deep sedation.

## 2. Materials and Methods

We did a search in the PubMed and Cochrane databases on 10 January 2024, with this strategy: (((“Cholangiopancreatography, Endoscopic Retrograde”[Mesh]) AND “Anesthesia”[Mesh]) OR “Moderate sedation”[Mesh]) OR “Deep Sedation”[Mesh].

We chose to limit our search to English-language articles and defined a restriction time between 2000 and 2024.

Exclusion criteria were studies on animals and children. Initially we proceeded to remove duplicates and then we carried out an initial screening based on the title and abstract. Relevant records were selected for review of full-text, and they were finally included if two investigators (MG and TVF) agreed on their eligibility.

**Figure 2:**
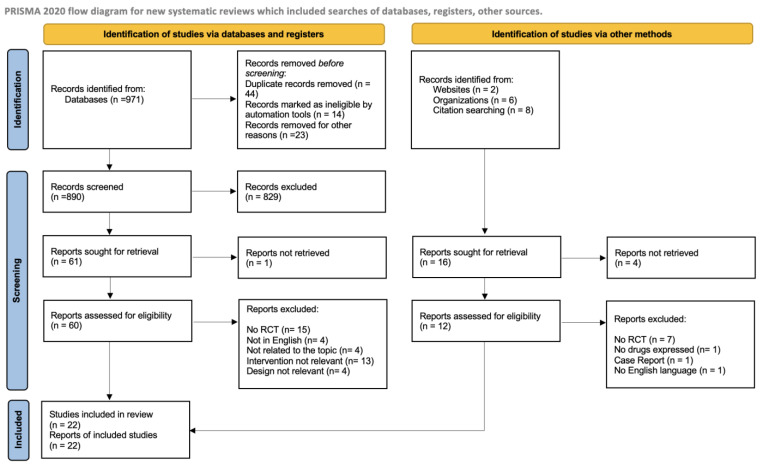
“PRISMA Flow Diagram”.

We also excluded abstract only records and conference proceedings. Following to the PRISMA recommendations checklist [4], the results will be presented in a narrative way, explaining and interpreting published evidence. Approximately 987 publications were retrieved, and 60 of them were selected, which included 48 original works. Articles were excluded based on the following criteria: not related to the topic, irrelevant design, not in English, and irrelevant intervention. Figure 2 shows the PRISMA-based flowchart for the selection of the studies.

## 3. What the Literature Tells Us

We carried out an open search in Pubmed® and The Cochrane Library for clinical trials in CENTRAL. The results are reported in Table 1. Several studies have evaluated the efficacy of dexmedetomidine (Dex) in comparison with other anesthetic drugs for gastrointestinal endoscopy [5]. 

Gupta and colleagues demonstrated that the tested doses of Dex were a safe and effective option for sedation and pain relief in patients undergoing procedures with monitored anesthesia care (MAC) and reduced the requirement for fentanyl and midazolam [6]. However, Jalowiecki and associates noted that the use of Dex for analgesia and sedation during outpatient colonoscopy is constrained by problematic side effects, notable hemodynamic instability, extended recovery times, and a complex administration process [7,8]. Furthermore, Hashiguchi et al. conducted a study to evaluate the safety and efficacy of Dex for the sedation of middle-aged patients having a conventional upper GI endoscopy [9]. Wu et al. carried out a prospective, randomized, double-blind trial to investigate the role of propofol against Dex on sedation during gastroscopy in outpatients [10]. Only two cases (5.9%) in the propofol group showed signs of respiratory depression. Both groups reported no significant changes in hemoglobin oxygen saturation (SpO2) or respiratory rate. According to the study, patients favored propofol injection for deeper sedation [10].

In their retrospective cohort analysis, Kim et al. examined the sedation efficacy of propofol or midazolam, concentrating on recovery time for different groups of patients [11]. The study found that utilizing low doses of propofol and midazolam together can allow physicians to avoid using opioids entirely and reduce propofol dosage [11].

In a cohort study, Amoros et al. reported that even deep sedation with propofol did not cause subclinical or overt hepatic encephalopathy in cirrhotic patients [12]. This observation was also confirmed by Riphaus et al. in a prospective, randomized study comparing propofol with midazolam for pre-endoscopic sedation in cirrhotic patients during upper gastrointestinal endoscopy [13]. Wahab et al. evaluated the use and handling of propofol vs. midazolam in patients, undergoing endoscopy, suffering from liver cirrhosis [14]. They examined 90 patients with compensated liver cirrhosis and reported that a low-dose combination of midazolam and propofol enhanced endoscopic outcomes including sedation and endoscopist satisfaction [14].

In the last ten years, the technique of sedation using Dex for the preservation of respiratory drive has gained popularity [6]. In a prospective randomized trial, Chen and colleagues examined the effectiveness of a single-loading dose of Dex combined with propofol for achieving deep sedation during ERCP in 49 elderly patients. Their findings showed that this approach decreases the need for propofol and mechanical airway support, while also offering better hemodynamic stability compared to propofol alone [15].

ERCP is more invasive than other endoscopic treatments since it includes drainage, stent implantation, and stone extraction [16]. In order to receive stable treatment, the patient must remain still [16]. On the other hand, increasing the amount of sedatives can cause airway obstruction as well as respiratory and circulatory depression associated with deep sedation.

Benzodiazepines (BZDs) can be used as an anesthetic during ERCP; however, BZDs sometimes cause paradoxical reactions such as disinhibition (e.g., unexpected or uncontrolled movements), making it difficult to perform an endoscopic procedure. In this regard, Ikeda et al. evaluated the usefulness of Dex combined with BZDs in patients who had difficulty in continuing ERCP due to BZD-induced disinhibition during the procedure [17]. The study, although it involved only 22 patients, shows that the movement score and the number of additional sedatives required were lower in the Dex group compared to the group of patients who had received only BZDs. Furthermore, a higher number of patients in the Dex group completed the procedure compared to patients in the BZDs-only group [17].

Koruk et al. conducted a study comparing the sedative and analgesic effects of midazolam–propofol versus Dex–propofol association, as well as their impact on hemodynamic and respiratory parameters in 40 adult patients undergoing ERCP [18]. Their findings showed that the Dex–propofol combination led to a shorter recovery time while producing similar sedation levels and side effects as the midazolam–propofol association. Furthermore, the study suggests that adding dexmedetomidine helps lower the required dose of propofol during the procedure [18].

Another important drug commonly used is Ketamine. Singh et al., in a prospective, single-blinded, randomized study on 84, ASA (American Society of Anesthesiologists) physical status I or II patients aged 18–65 years presenting for ERCP in a tertiary care center, have compared the combination of ketamine–Dex (KD) with the combination of ketamine–propofol (KP) [19]. The authors described that the mean SpO2 in the KP group was significantly lower than the KD group and that the lowest mean arterial pressure and heart rate in the KD group were significantly lower than in the KP group [19]. The study concluded that the combination of KD is a safe alternative to KP with a lower risk of respiratory complications [19].

Pushkarna et al. in their study evaluated the role of propofol using midazolam and Dex as premedication for ERCP, showing that Dex provided intense and better sedation quality along with lesser requirement of propofol doses [20].

In 2014 Sethi et al. presented an interesting open-label RCT comparing midazolam and Dex [21]. The authors highlighted that Dex may be a better solution compared to the use of midazolam for moderate sedation in ERCP. In fact, this is characterized by an early recovery, a better patient and endoscopist satisfaction score, fewer complications and a better facial pain scale score at the end of the procedure [21].

In a prospective, comparative study published in 2017, the authors compared the safety of midazolam versus propofol for ERCP sedations realized by non-anesthesiologists in 100 older patients [22]. Main outcome measurements were incidents of cardiopulmonary events and efficacy measured on a 10-point visual analogue scale (VAS). No significant differences were reported between the two groups of patients studied [22].

Dex is a safe medication used to treat the elderly and challenging patients. In fact, Inatomi et al. undertook a retrospective study to value the use of Dex sedation during ERCP in very old patients (>80 years) [23]. The study shows that Dex can decrease the incidence of respiratory complications and the total dose of other sedative agents [23].

Lu et al. compared the use of the combination Dex–remifentanil (DR) with a combination midazolam–remifentanil (MR) for sedation during ERCP. Their findings indicated that the DR protocol provided comparable sedative efficacy while offering improved respiratory protection [24]. Additionally, higher patient satisfaction scores suggest the potential for enhanced reproducibility in ERCP quality. The use of Dex combined with remifentanil is considered a safe option for moderate sedation during ERCP [24].

Mukhopadhyay and colleagues assessed the effectiveness of Dex as an additional agent for extended deep sedation during ERCP. They compared three different deep sedation protocols in terms of safety and effectiveness for prolonged therapeutic ERCP procedures [25]. The study demonstrates that a combination of anesthetic drugs resulted in a better outcome than the conventional propofol–midazolam regimen and that Dex as an additional therapy increases the efficacy and safety of the sedative–analgesic effect. Additionally, Dex reduces the amount of propofol used and helps keep the patient at a safe and more stable level of sedation. It also increases the anesthesiologist’s satisfaction and perception of safety and tranquility [25].

In a randomized controlled trial conducted by Lee et al., the researchers evaluated and compared the sedative effects and adverse events of two regimens during ERCP: midazolam–meperidine–dexmedetomidine (MMD) versus midazolam–meperidine. The study concluded that incorporating Dex into the midazolam–meperidine combination resulted in enhanced sedative effectiveness and improved hemodynamic stability during the procedure, compared to using midazolam–meperidine alone [26].

Goyal et al. compared the efficacy and safety of a standard propofol–fentanyl (PF) regimen with a Dex and ketamine (KD) combination [27]. Hemodynamic stability and intraoperative peripheral venous saturation was better maintained with Dex and ketamine, but hospital length of stay was shorter with propofol and fentanyl [27].

A total of 134 patients with ASA classification I–III were included in a RCT that compared satisfaction, recovery score, and recovery/safety profiles for ERCP sedation between continuous infusion of propofol and another generic type of sedation [28]. The study concluded that for the maintenance of a good level of sedation it is necessary to have well trained personnel [28].

In recent years, the use of remimazolam, a new ultrafast-acting benzodiazepine, has become widespread. Two other studies compared the use of remimazolam and propofol for inpatient ERCP. In one of these (Lee et al.), of the 110 patients randomized, 108 underwent sedation and ERCP; in particular, 53 patients received remimazolam and 55 received propofol [27]. Patients receiving propofol initiated ERCP earlier than patients receiving remimazolam. Time to full alertness after ERCP was also significantly shorter in the propofol arm [29].

Zhang et al., in a RCT that included 99 patients undergoing elective ECRP, compared the use of meperidine/midazolam, remifentanil, or remifentanil plus midazolam [30]. The blood pressure, heart rate, respiratory rate, O_2_-saturation, and bispectral index (BIS) values of the patients were recorded. Hypoxemia was observed most frequently in patients who received remifentanil plus midazolam and an significant increase of blood pressure was observed in patients treated with meperidine/midazolam and with remifentanil [30].

Dong’s study involved the randomization of 518 patients, with 250 assigned to the remimazolam group and 255 to the propofol group. During ERCP, 9.6% of patients in the remimazolam group experienced hypoxia, compared to 15.7% in the propofol group. The need for airway intervention due to hypoxia was notably higher in the propofol group [31]. Furthermore, patients who received remimazolam had fewer episodes of cardiovascular instability than patients sedated with propofol [31]. Patients receiving remimazolam sedation were satisfied and stated that they would like to reuse this drug for any future interventions [31].

A prospective, double-blind, randomized, controlled clinical trial (Wang P et al.), conducted from May 2018 to June 2019, randomly divided 400 patients into two groups using a computer-generated randomization table; one group received propofol and nalbuphine (PN) and the other group received propofol and fentanyl (PF) [32]. Respiratory depression was the primary outcome and surgical interruptions was the secondary outcome in the study. Patients in the PN group had fewer episodes of respiratory depression than patients in the PF group. Fourteen patients developed marked hypoxia in the PF group, while only six developed it in the PN group [32]. The study demonstrated that sedation using a PN combination for ERCP significantly reduced the occurrence of respiratory depression and surgical interruptions when compared to sedation with a PF combination. Furthermore, the author had observed no differences in the manifestation of postoperative pain and cardiocirculatory stability between the two groups [32].

Guo et al. analyzed the use of oxycodone combined with propofol versus fentanyl combined with propofol. A total of 193 patients aged 65 to 80 years undergoing ERCP were divided into two groups: an “oxycodone combined with propofol” group (OP group, n = 97) and a “fentanyl combined with propofol” group (FP group, n = 96) [33]. The authors found that there were no differences in the manifestation of hypotension or bradycardia between the two groups, but there were more episodes of desaturation (SpO2 < 90% for >10 s in 8.3%), postoperative nausea (7.3%), and vomiting (5.2%) in the FP group compared to the OP group [33].

A randomized, double-blind, noninferiority trial conducted at a single center involved patients with an American Society of Anesthesiologists (ASA) physical status of I to II, all of whom were scheduled for ERCP. Patients were assigned to receive either etomidate or propofol, depending on their group allocation, with the primary focus being the occurrence of any respiratory event. Respiratory complications were observed in 10 patients (15.6%) in the etomidate group and 16 patients (25.4%) in the propofol group [34]. The study highlights how more cardiovascular events occurred in the etomidate group [34].

In a clinical trial by Akhondzadeh et al. the use of propofol–fentanyl (PF) was compared with propofol–ketamine (PK) to sedate patients undergoing ERCP [35]. The two groups were found to be comparable for episodes of cardiocirculatory instability. Although post-procedural pain was less in the PK group in comparison to the PF group, it was not statistically significant [35]. They also assessed the sedation score between the two groups and found no significant difference between the two groups. There was no significant difference in the total dose of propofol use between the two groups.

In a single center RCT, 37 patients undergoing ERCP received either conventional sedation with midazolam and pethidine or a combination of midazolam and ketamine [36]. The authors studied and compared between the two groups the depth of sedation. Ketamine may have potential as an agent for sedation in higher risk patients [36].

The study by Haytural et al. aimed to evaluate the impact of using propofol alone, a combination of propofol and remifentanil, and a combination of propofol and fentanyl on the total required dose of propofol during ERCP, as well as on post-procedure pain scores. This randomized trial included 90 patients aged 18 to 70 years, with ASA physical statuses of I, II, or III, who underwent sedation and analgesia for elective ERCP. Group I received only propofol (1.5 mg/kg), Group II received a combination of remifentanil (0.05 μg/kg) and propofol (1.5 mg/kg), and Group III was given a combination of fentanyl (1 μg/kg) and propofol (1.5 mg/kg). Sedation levels for all patients were evaluated using the Ramsey Sedation Scale (RSS) [37]. No statistically significant differences were detected between the groups in terms of changes in systolic arterial pressure, diastolic arterial pressure, mean arterial pressure, and saturation levels throughout the follow-up. (*p* > 0.05). They also assessed the effects of drugs and the change of hemodynamics lead to changes in monitorization time [37].

In a single-center, observational, prospective, cohort study, 106 patients underwent ERCP. Deep sedation in spontaneous breathing was achieved by intravenous administration of propofol and remifentanil [38]. Only two patients out of 106 had to change the anesthesiological plan and resort to general anesthesia following the onset of bronchial reactivity, cough, and desaturation, probably deriving from difficult endoscopic maneuvers. Among the minor adverse reactions recorded, only 3% of patients showed hypotension and 2% desaturation [38].

Breazu et al. in a prospective, randomized, double-blind, placebo-controlled study that was conducted between February 2022 and April 2022 in Romania with 83 patients over 65-year old, with ASA scores of II–IV, undergoing an ERCP procedure, compared the use of lidocaine plus propofol and saline solution plus propofol [39]. The study showed that the combination with lidocaine reduces the dose of propofol. However, the postprocedural pain did not differ between the two groups [39].

### The Characteristic of Anesthetic Agents

Dex, a selective alpha-2-agonist with a short half-life, has anxiolytic, hypnotic, and analgesic effects [40]. It is eight times more selective for the alpha-2 adrenergic receptor than clonidine, as well as 1620 times more potent as an alpha-2 adrenergic receptor agonist than an alpha-1 adrenergic receptor agonist. Scientific evidence has shown that Dex produces analgesia, anxiolysis, and sedation in a dose dependent manner without respiratory depression [40]. It has a sympatholytic effect through inhibition of norepinephrine release in sympathetic nerve endings. In addition to causing moderate sedation of the patient, Dex causes a reduction in heart rate and blood pressure through a central sympatholytic action. It should be underlined, however, that at high concentrations it can cause peripheral vasoconstriction [40,41].

It is important to have caution if you decide to administer Dex to patients with chronic bradycardia. Scientific evidence on the effects of Dex in patients with heart rates lower than 50–55 is very limited. Dex-induced bradycardia usually does not require treatment but responds well to a reduction of the dose of the drug and, if necessary, administration of anti-cholinergic drugs [41] [Table 2].

Midazolam is a hypnotic-sedative drug with anxiolytic, muscle relaxant, anticonvulsant, sedative, hypnotic, and amnestic effects. It is a part of the benzodiazepine class [42]. This drug is distinguished from others in its class by its quick onset of action and medium duration of activity. Midazolam is available orally, rectally, intranasally, intramuscularly, and intravenously. This drug was initially approved by the US Food and Drug Administration (FDA) in 1985 and has subsequently gained approval for various indications. It has many fields of application: pre-anesthesia, sedation, general anesthesia, epilepsies, etc. It has an immediate sedation and anterograde amnesia action. Occasionally, especially in elderly subjects, its disposal may be slowed down. It is also used as a drug to induce unconsciousness in hemodynamically unstable subjects, precisely because the drug causes minimal cardiorespiratory depression. Midazolam was first synthesized in 1976 by Fryer and Walser [42] [Table 2].

Propofol is a short-acting hypnotic agent that is administered intravenously. The appearance of propofol is milky (similar to that of some fat emulsions used for parenteral nutrition), as it contains soybean oil and egg yolk lecithin [43]. This, together with the amnesia effects, is why propofol is also colloquially called “Milk of amnesia”. The molecule is characterized by its high lipophilicity, therefore it tends to distribute rapidly in all the biological tissues of the organism, as well as in the central nervous system. The molecule is excreted by the body mainly via the urine, as sulphate or glucuronide derivatives. Less than 2% of a dose is eliminated in the feces. After intravenous infusion, the elimination half-life varies between 277 and 403 min. Propofol is believed to have different mechanisms of action. In particular, it seems to be able to determine a positive modulation of the inhibitory function of gamma-aminobutyric acid (GABA) through the GABA A receptors, thus slowing down the closing time of the channel, and it also acts as a blocker of sodium channels [43] [Table 2].

Ketamine is an analgesic-dissociative drug, the only compound of the arylcyclohexylamine class approved for medical use, and is used for induction and maintenance of anesthesia. Ketamine is reported by the World Health Organization in its List of Essential Medicines; a list that lists the effective and safe medicines essential to a hospital. It has been authorized in the US as a general dissociative anesthetic since the 1970s. Ketamine exhibits a general anesthetic action of the non-barbiturate type with a rapid rate of action. In humans, when administered intravenously and at a dosage of 1 mg/kg, it causes analgesia and anesthesia within 30 s; the anesthetic state lasts for a time ranging from 3 to 25 min and does not lead to respiratory depression or alteration of airway reflexes. As evidenced also through experimental animals, it produces a mostly cataleptic and anesthetic action rather than a sedative and hypnotic one [44]. Ketamine therefore determines a dissociative anesthesia, as it has depressive effects on the thalamo–cortical system and activates the limbic system and reticular formation. The analgesic and anesthetic action of ketamine has been related to its NMDA-receptor-blocking activity. It is hypothesized that the same mechanism underlies the antidepressant effect of the molecule [44] [Table 2].

Fentanyl is a powerful opioid analgesic that can be used as an analgesic adjunct to general anesthesia or as a stand-alone anesthetic drug. Fentanyl exhibits µ-agonist properties. The agonist behavior towards the δ- and κ-receptors is comparable to that of morphine [45]. Fentanyl is mainly metabolized in the liver. The drug undergoes high first pass clearance. Elimination occurs mainly via the urine in the form of metabolites and only about 10% as the unchanged drug [45]. Fentanyl exhibits triphasic plasma kinetics with a half-life of approximately 3.7 h; following intravenous injection, plasma fentanyl concentrations decline rapidly, with a sequential distribution half-life of approximately 1 min and 18 min and a terminal elimination half-life of 475 min. The plasma protein binding of fentanyl is approximately 84%. Fentanyl is rapidly metabolized, mainly in the liver by CYP3A4. The major metabolite is norfentanyl. The clearance of fentanyl is 574 mL/min. Approximately 75% of the administered dose is excreted in the urine within 24 h, of which only 10% is eliminated as unchanged drug [45] [Table 2].

Remifentanil is a short-acting mu-opioid agonist. Structurally it belongs to the same family as fentanyl and other phenylpiperidines but differs from fentanyl both in its pharmacokinetics and in its metabolism: remifentanil undergoes extrahepatic metabolism by non-specific plasma and tissue esterases [46]. For these reasons, the time required for reduction of any percent plasma concentration of remifentanil after discontinuation of the infusion is independent of the duration of the infusion. The pharmacokinetic profile of remifentanil is organ independent and the dosage should be adjusted only in elderly patients by reducing the bolus and infusion dosage and in obese subjects by calculating the intravenous dosages according to age and ideal weight [46]. Remifentanil causes both a reduction in the MAC of halogenated anesthetics and a decrease in other anesthetic drugs requirements. Remifentanil can be used for tracheal intubation without muscle relaxants; for the management of sedation, also in association with midazolam and/or with propofol; furthermore, as an analgesic for monitored anesthesia care in critically ill patients in intensive care; and for postoperative analgesia when an appropriate analgesic strategy has not been planned [46] [Table 2].

Ciprofol is a short-acting intravenous sedative based on the structural modification of propofol. It is a compound similar to propofol in chemical structure and hypnotic effect [47]. This drug has high efficacy, good selectivity, and fewer adverse reactions, indicating good clinical application potential. Ciprofol adds a cyclopropyl group to the side chain of the core structure. The addition of this crucial structure reduces the lipophilicity of the parent structure by increasing the spatial effect [47]. Like propofol, ciprofol is a positive allosteric modulator and direct agonist of the GABA A receptor. The higher selective binding ability of ciprofol to the receptors enables it to achieve the same sedative and anesthetic effects as propofol at a lower dosage. The safety advantage of ciprofol may provide a more stable anesthesia process for the clinic and effectively reduce and alleviate postoperative complications in patients, especially in the elderly [47].

Remimazolam is a fast-acting, ultra-short-acting benzodiazepine used primarily for sedation in various medical procedures [48]. It provides rapid onset of sedation and a quick recovery time, making it suitable for outpatient settings. The drug is metabolized by tissue esterases, leading to a predictable pharmacokinetic profile [48]. Due to its safety and effectiveness, remimazolam is gaining popularity in anesthesia and procedural sedation. Remimazolam is administered intravenously and is associated with rapid onset and offset of sedation. In clinical trials, peak sedation occurred 3 min after the initial bolus and patients were fully awake 12–14 min after the last dose of it [48].

**Table 2:** The main findings of the included trials.

No.	First Author	Year	Type of Study	Population Undergoing ERCP	Intervention	Comparator
1	Chen M. [15]	2022	Prospective, randomized trial.	49 patients	Dexmedetomidine group	Propofol (PRO group)
2	Ikeda I. [17]	2022	Retrospective, single-center study.	22 patients	Benzodiazepine (BZD group)	Dexmedetomidine group
3	Koruk S. [18]	2020	Randomized, prospective, double-blind study.	40 patients	Midazolam + propofol group	Dexmedetomidine + propofol group
4	Singh A. [19]	2022	Prospective, single-blinded randomized study.	84 patients	Ketamine + dexmedetomidine (Keto–Dex group)	Ketamine + propofol (Keto–Fol group)
5	Pushkarna G. [20]	2019	Randomized, assessor-blinded study	60 patients	Dexmedetomidine as premedication to propofol anesthesia group	Midazolam as premedication to propofol anesthesia group
6	Sethi P. [21]	2014	Open-label, randomized, controlled trial	60 patients	Dexmedetomidine group	Midazolam group
7	Inatomi O. [23]	2018	Retrospective, single-center study.	62 patients	Dexmedetomidine group	Midazolam group
8	Lu Z. [24]	2018	Prospective, randomized, single-blinded, preliminary trial.	198 patients	Dexmedetomidine + Remifentanil (DR group)	Midazolam + Remifentanil (MR group)
9	Mukhopadhyay S. [25]	2015	Prospective, randomized, controlled, assessor-blinded study.	45 patients	Propofol + Midazolam group	(1) Ketamine–Propofol–Midazolam–Pentazocine group (2) Ketamine–Propofol–Midazolam–Pentazocine–Dexdemedetomidine group
10	Lee B.S. [26]	2014	Prospective, randomized, double-blinded trial.	110 patients	Midazolam, meperidine, dexmedetomidine group	Midazolam + meperidine group
11	Goyal R. [27]	2016	Randomized, controlled trial.	83 patients	Dexmedetomidine + ketamine (DK group)	Propofol + fentanyl (PF group)
12	Lee J. [29]	2023	Randomized, single-blind, single-center study.	110 patients	Remimazolam group	Propofol group
13	Dong S.A. [31]	2023	Randomized, controlled, clinical trial.	518 patients	Remimazolam + alfentanil group	Propofol + alfentanil group
14	Breazu C.M. [39]	2022	Randomized, controlled trial.	83 patients	Lidocaine and propofol (L group)	Saline solution and propofol (C group)
15	Wang P. [32]	2022	Randomized, controlled trial.	400 patients	Propofol + nalbuphine (PN group)	Propofol + fentanyl (PF group)
16	Guo P. [33]	2022	Randomized, controlled trial.	193 patients	Oxycodone + propofol (OP group)	Fentanyl + propofol (FP group)
17	Park C.H. [34]	2018	Randomized, controlled trial.	127 patients	Etomidate group	Propofol group
18	Han S.J. [22]	2017	Randomized, controlled trial.	100 patients over 80 years of age	Midazolam + fentanyl (MF group)	Propofol + fentanyl (PF group)
19	Zhang J. [30]	2016	Randomized, controlled trial.	99 patients	Meperidine + midazolam (C group)	(1) Remifentanil (R group) (2) Remifentanil + midazolam (RM group)
20	Akhondzadeh R. [35]	2016	Randomized, controlled trial.	98 patients	Propofol + ketamine (PK group)	propofol–fentanyl (PF group)
21	Haytural C. [37]	2015	Randomized, controlled trial.	90 patients	Propofol group	(1) Remifentanil + propofol group (2) Fentanyl + propofol group
22	Narayanan S. [36]	2015	Randomized, controlled trial.	37 patients	Midazolam + ketamine group	Midazolam +pethidine group
23	Kongkam P. [28]	2008	Randomized, controlled trial.	134 patients	Propofol group	Meperidine + midazolam group
24	Barnett S.R. [49]	2013	Prospective, observational study.	438 patients	Deep sedation (ADDS) in non-intubated patients	General endotracheal anesthesia (GET)
25	De Vico P. [38]	2023	Prospective, observational, single-center, cohort study.	106 patients	Deep sedation with an association of propofoland and remifentanil	General anesthesia (GA)

## 4. Discussion

The level of sedation and airway management during an invasive procedure is often based upon the patient’s safety and comfort, the patient’s comorbidities, the type and the degree of invasiveness of the procedure, the duration of the procedure, and the sedation level that needs to be achieved in order to facilitate the procedure.

In recent years, numerous studies and documents have confirmed the safety of unintubated ERCP [1,50]. *Goudra* et al. examined 653 patients who had elective ERCP procedures at the authors’ outpatient center [51]. They observed that most of the procedures can be conducted without endotracheal intubation (intubation rate was <1%) and observed that endotracheal intubation was performed only in cases with risk factors for aspiration [51]. There were no interruptions or emergency endotracheal intubations. Even in their inpatient endoscopy center, where patients were significantly more severe, the incidence of endotracheal intubation was <10% [51]. In other studies, roughly 10% of non-obese individuals undergoing ERCP required endotracheal intubation.

*Garewal* et al., in their review, evaluated and compared the safety and effectiveness of conscious sedation, based on the use of midazolam and meperidine, with deep sedation, based on the administration of propofol [52]. The authors concluded that there is evidence that patients who received propofol had a more rapid hospital admission. From a safety point of view, however, the anesthetics were comparable [52].

In an observational study, the safety profile of deep sedation compared with general anesthesia with orotracheal intubation was prospectively evaluated [49]. A total of 393 patients received deep sedation and 45 patients received general anesthesia. During the procedure, for 16 (3.7%) patients in deep sedation, it was necessary to proceed with orotracheal intubation and to start general anesthesia due to intraoperative complications [49].

The principle objective of a sedation technique is to lessen a patient’s worry and agitation while also improving their tolerance for the procedure. Analgosedation can be divided into four types: minimal (anxiolysis), moderate (moderate sedation), deep sedation, and general anesthesia. Deep sedation is identified as a state in which a patient cannot be awoken easily but responds consciously to repeated painful stimuli.

The choice to perform deep sedation or moderate sedation can be made as part of the anesthetic preassessment process and will be influenced by protocols, guidelines, and experience of the team [50]. Patients who desire deep sedation or anesthesia should be pre-assessed and the American Society for Gastrointestinal Endoscopy (ASGE) suggest that ‘all patients should be assessed for risk of sedation related adverse effects’. In patients with a suspected-difficult airway, it is necessary to evaluate the risks of deep sedation [53].

Analgosedation, in addition to having the objective of improving patient comfort, also allows for improving the performance of the endoscopist, and therefore improves the outcome of the procedure. However, the anesthesiologist’s task is to prevent respiratory depression due to sedation from leading to a suspension or slowing down of the procedure. Deep sedation must be distinguished from moderate sedation within the context of anesthesia. This is the recommended level of sedation for patients undergoing elective endoscopy. Deeper sedation than intended is associated with a higher likelihood of complications [53,54,55].

The guidelines established in 2002 by the “*American Society of Anesthesiologists (ASA) task force*” on sedation and analgesia by non-anesthesiologists, which have also been endorsed by the *American Society for Gastrointestinal Endoscopy* and the “Guidelines for sedation and anesthesia in Gastrointestinal endoscopy” prepared by the Practice Committee of the *American Society for Gastrointestinal Endoscopy (ASGE)*, suggest that all patients should be pre-assessed for risk of sedation-related adverse effects and the provider should have training in diagnosing and managing sedation-related adverse events [50,53].

## 5. Limitations

This study has some limitations. In fact, we only explored the PubMed and Cochrane databases; in the future, exploration and analysis of other databases could help us identify new studies and new evidence in this area. A step forward could be represented by a systematic review and meta-analysis with analysis of multiple databases.

## 6. Conclusions

In conclusion, based on this review of the literature, it seems that Dex is associated with better sedation for gastrointestinal endoscopic procedures, including a reduction of respiratory complications when compared with other anesthetics.

If moderate sedation is performed, the patient is able to maintain protective airway reflexes and can recover quickly. A faster recovery of the patient at the end of the endoscopic procedure is an advantage for both the patient and hospital.

There is no evidence of a correlation between the drug administered and the onset of cardiopulmonary complications and there are no data demonstrating that in case of administration together with other anesthetics, recovery times may increase.

However, it is not possible to generalize or express a univocal conclusion as each patient has a unique clinical history.

Although difficult, further prospective studies could support the role of these “tailored” sedation strategies in patients who undergo endoscopic retrograde cholangiopancreatography (ERCP).

## Figures and Tables

**Table 1:** Characteristics of anesthetic drugs.

Drug	Pharmacological Class(s)	Use:	Metabolism	Excretion
Ciprofol	- γ-Aminobutyric acid agonist - Hypnotic agent	Intravenous	Liver metabolism: glucuronidation at the C1-hydroxyl.	Urine
Dexmedetomidine	Alpha-2-adrenergic agonist	Intravenous: bolus dose + maintenance dose	Liver metabolism: - Direct N-glucuronidation, - Direct N-methylation; - Oxidation catalyzed by cytochrome P450.	Urine
Etomidate	Short-acting intravenous anesthetic agent	Intravenous	Liver Metabolism: Metabolized rapidly by ester hydrolysis to inactive metabolites.	Urine
Ketamine	NMDA receptor antagonist	Intravenous	Liver metabolism: - N-dealkylation; - Hydroxylation of the cyclohexone ring - Conjugation to glucuronic acid; - Dehydration of the hydroxylated metabolites for the formation of cyclohexene derivatives.	Urine: - 2% is excreted unchanged; - 2% in the form of norketamine; - 16% as dehydronorketamine; - 80% as conjugates of hydroxylated ketamine metabolites with glucuronic acid.
Fentanyl	Mu-Type Opioid Receptor agonist	Intravenous	*Liver metabolism:* oxidative N-dealkylation into norfentanyl at the piperidine ring by hepatic CYP3A4 and 3A5 isoenzymes.	- 75% Urine - 9% Feces
Lidocaine	Local Anesthetic	Intravenous Spray	Liver Metabolism: - De-ethylation to monoethylglycinexylidide (MEGX); - De-ethylation to glycinexylidide and hydrolysis to 2,6-xylidine.	Urine
Meperidine	Mu-Type Opioid Receptor agonist	Intravenous	*Liver metabolism:* - hydrolysis to meperidinic acid followed by partial conjugation with glucuronic acid; - N-demethylation to normeperidine.	Urine
Midazolam	Benzodiazepines	- Intravenous - Nasal use - Rectal use	*Liver metabolism:* hepatic microsomal enzyme cytochrome P450 (CYP) 3A4.	Urine
Nalbuphine	Synthetic opioid agonist-antagonist	Intravenous	Liver Metabolism: metabolized in the liver to inactive glucuronide conjugates.	Urine
Oxycodone	Opioid analgesic drugs	Intravenous	Liver Metabolism: metabolized by CYP3A4/5, which mediates the N-demethylation of oxycodone to noroxycodone.	Urine
Propofol	Intravenous hypnotic anesthetic agent	Intravenous	Liver metabolism: glucuronidation at the C1-hydroxyl.	Urine
Remifentanil	Mu-Type Opioid Receptor agonist	Intravenous	Metabolism independent of organ function. It is metabolized by non-specific plasma esterases.	Urine
Remimazolam	Ultra-short-acting intravenous benzodiazepine	Intravenous	Tissue esterase	Urine
